# Uncovering vector, parasite, blood meal and microbiome patterns from mixed-DNA specimens of the Chagas disease vector *Triatoma dimidiata*

**DOI:** 10.1371/journal.pntd.0006730

**Published:** 2018-10-18

**Authors:** Lucia C. Orantes, Carlota Monroy, Patricia L. Dorn, Lori Stevens, Donna M. Rizzo, Leslie Morrissey, John P. Hanley, Antonieta Guadalupe Rodas, Bethany Richards, Kimberly F. Wallin, Sara Helms Cahan

**Affiliations:** 1 Rubenstein School of Environment and Natural Resources, University of Vermont, Burlington, Vermont, United States of America; 2 Laboratorio de Entomología Aplicada y Parasitología, Escuela de Biología, Universidad San Carlos de Guatemala, Ciudad de Guatemala, Guatemala; 3 Department of Biological Sciences, Loyola University New Orleans, New Orleans, Louisiana, United States of America; 4 Department of Biology, University of Vermont, Burlington, Vermont, United States of America; 5 Department of Civil and Environmental Engineering, University of Vermont, Burlington, Vermont, United States of America; 6 USDA Forest Service, Northern Research Station, Burlington, Vermont, United States of America; Instituto de Ciências Biológicas, Universidade Federal de Minas Gerais, BRAZIL

## Abstract

Chagas disease, considered a neglected disease by the World Health Organization, is caused by the protozoan parasite *Trypanosoma cruzi*, and transmitted by >140 triatomine species across the Americas. In Central America, the main vector is *Triatoma dimidiata*, an opportunistic blood meal feeder inhabiting both domestic and sylvatic ecotopes. Given the diversity of interacting biological agents involved in the epidemiology of Chagas disease, having simultaneous information on the dynamics of the parasite, vector, the gut microbiome of the vector, and the blood meal source would facilitate identifying key biotic factors associated with the risk of *T*. *cruzi* transmission. In this study, we developed a RADseq-based analysis pipeline to study mixed-species DNA extracted from *T*. *dimidiata* abdomens. To evaluate the efficacy of the method across spatial scales, we used a nested spatial sampling design that spanned from individual villages within Guatemala to major biogeographic regions of Central America. Information from each biotic source was distinguished with bioinformatics tools and used to evaluate the prevalence of *T*. *cruzi* infection and predominant Discrete Typing Units (DTUs) in the region, the population genetic structure of *T*. *dimidiata*, gut microbial diversity, and the blood meal history. An average of 3.25 million reads per specimen were obtained, with approximately 1% assigned to the parasite, 20% to the vector, 11% to bacteria, and 4% to putative blood meals. Using a total of 6,405 *T*. *cruzi* SNPs, we detected nine infected vectors harboring two distinct DTUs: TcI and a second unidentified strain, possibly TcIV. Vector specimens were sufficiently variable for population genomic analyses, with a total of 25,710 *T*. *dimidiata* SNPs across all samples that were sufficient to detect geographic genetic structure at both local and regional scales. We observed a diverse microbiotic community, with significantly higher bacterial species richness in infected *T*. *dimidiata* abdomens than those that were not infected. Unifrac analysis suggests a common assemblage of bacteria associated with infection, which co-occurs with the typical gut microbial community derived from the local environment. We identified vertebrate blood meals from five *T*. *dimidiata* abdomens, including chicken, dog, duck and human; however, additional detection methods would be necessary to confidently identify blood meal sources from most specimens. Overall, our study shows this method is effective for simultaneously generating genetic data on vectors and their associated parasites, along with ecological information on feeding patterns and microbial interactions that may be followed up with complementary approaches such as PCR-based parasite detection, 18S eukaryotic and 16S bacterial barcoding.

## Author summary

Chagas disease is caused by the parasite *Trypanosoma cruzi*, which is spread by triatomine kissing bugs. There are many biotic factors that influence the risk of disease transmission, including the strain of the parasite, the vector movement patterns, the community of microbes interacting with the parasite inside the vector's gut, and the availability of suitable vertebrate hosts. DNA from all of these species can be found in the gut of an infected bug, providing an opportunity to investigate all of them simultaneously by genetically analyzing this single tissue. In this study, we developed a DNA-based method to retrieve, separate, and analyze genetic information from the abdomens of 32 *T*. *dimidiata* kissing bug vectors collected across Central America. We found two distinct strains of *T*. *cruzi*, and four *T*. *dimidiata* genetic clusters associated with environmental and geographical characteristics. These populations harbored different bacterial gut communities that were augmented by specifically infection-associated bacteria when the vector was infected by the parasite. In some cases, we could identify what the insect had recently fed on, including chicken, duck, dog and human. Having simultaneous information on all of these organisms may help to fine-tune control strategies that influence the risk of *T*. *cruzi* transmission.

## Introduction

Chagas disease (American trypanosomiasis) is caused by the protozoan parasite *Trypanosoma cruzi*. Considered a neglected disease by the World Health Organization, it is widespread in the Americas, where an estimated 70 million people are at risk of contracting the infection [1]. The disease is most prominent in poor, rural communities of South and Central America, where the disruption of sylvatic ecosystems and precarious socioeconomic conditions aid the establishment of domestic and peridomestic vector populations [1,2, 3].

The infective agent, *Trypanosoma cruzi*, is genetically diverse and widely dispersed in the Americas [4, 5, 6, 7]. Multiple strains are distributed from the southern United States to northern Argentina, and are ancestrally linked to sylvatic and/or domestic transmission cycles depending on their habitat affiliation [4, 8, 9]. From an epidemiological standpoint, *T*. *cruzi sensu lato* (s.l.) is the most important group of parasitic trypanosomes strains, comprising *T*. *cruzi cruzi*, which causes Chagas disease in humans, and *T*. *cruzi marinkellei*, a strain uniquely found in South American bats [5, 10, 11]. Within *T*. *c*. *cruzi*, seven Discrete Typing Units (DTUs) have been characterized (TcI-VI and TcBat) [4, 11, 12, 13]. All DTUs can cause disease in humans; however, their relative abundance varies among ecological and geographical niches, and they show variation in clinical epidemiology and prevalence in domestic ecotopes [12]. TcI is the predominant DTU in the Americas, found in arboreal *Rhodnius* species from Central America to Ecuador, and in sylvatic and domestic *Triatoma* from the southern United States to northern Argentina [4, 5, 13]. It is also reported in other Triatominae genera such as *Meccus*, *Mepraia* and *Panstrongylus*, and its genetic diversity is consistent with its long evolution in the continent, dating between 3–4 MYA [4, 14]. TcIV, a DTU hypothesized as an ancestral hybrid between TcI and TcII, is the only other DTU that has been detected in vector and human specimens in Central America [4, 13, 15]. Although there are 84 reports of humans infected with TcIV from six countries, there is evidence that this DTU is of sylvatic origin and exclusively associated with sylvatic vectors [4].

In addition to *T*. *cruzi* diversity, the genetic structure of the vector, driven by geographical and ecological factors, is also likely to play an important role in determining human infections. To date, more than 140 species of New World triatomines have been described [16, 17, 18] and a small number of species have been reported from Asia. The majority are associated with sylvatic habitats, but species such as *Triatoma infestans* and *Rhodnius prolixus* have adapted to domestic and peridomestic niches [7, 16, 19, 20, 21, 22, 23]. Furthermore, species like *T*. *dimidiata* are in the process of domiciliation, establishing multi-generational colonies in human households, therefore increasing the risk of *T*. *cruzi* transmission to humans [23]. In Central America, *R*. *prolixus* was the predominant Chagas disease vector until successful eradication of the vector in 2010 [21]. In its place, endemic triatomines including *T*. *dimidiata* have colonized vacant peridomestic and domestic habitat niches and have slowly changed the dynamics of disease transmission in these ecotopes [24, 25, 26, 27, 28]. *Triatoma dimidiata* is widely distributed from Mexico to Perú in sylvatic, peridomestic and domestic habitats [26, 29, 30]. It is morphologically highly variable across this range, with phenotypic variation among sylvatic and domestic ecotopes, as well as geographical niches [23, 30]. Population genetic analyses using various molecular markers have yielded conflicting assessments of the extent and importance of genetic structuring across its geographical distribution; nevertheless, most studies agree that it is genetically diverse [17, 24, 26, 27, 29, 31].

The microbial community colonizing the vector’s gut may further influence parasite transmission to vertebrate hosts. When the parasite is ingested in a blood meal, the parasite moves into the midgut, where availability of glucose moderates its transformation to replicative epimastigotes [32, 33]. In the midgut, the parasite attaches to the cuticle wall prior to differentiating into a metacyclic form [33]. Although the composition and physiological role of gut bacteria in triatomines are largely unknown, bacterial communities can significantly modify glucose levels in anaerobic environments such as the gut, facilitating or impeding colonization of the insect’s digestive tract by pathogens such as *T*. *cruzi* [34, 35, 36, 37]. Some bacterial species have been shown to directly inhibit colonization by *T*. *cruzi* in *Triatoma* and *Rhodnius* spp. (e.g., *S*. *marecescens*) [35, 38], either in their native form, or as introduced transgenics in the gut of triatomines under laboratory conditions [39, 40, 41]. At the same time, *T*. *cruzi* infection may be capable of decreasing the microbial population in the gut and modifying the nitrite/nitrate production important for triggering defense metabolic cascades [42].

As a vector-borne disease, domestic and sylvatic transmission cycles are dependent on the diversity and availability of vertebrates, both as blood meals for the vector and as potential hosts [43]. *Trypanosoma cruzi* is most commonly transmitted to mammalian hosts via contamination of a wound or mucous membrane by the parasite-contaminated feces of the vector, and/or by direct ingestion of an infected insect [5, 33]. In domestic ecotopes, humans and dogs are presumed to serve as both the primary blood meals of the vector and the main mammalian source of the parasite; however, there are numerous peridomestic hosts (e.g. small ruminants, rodents, pigs) that may be important contributors to disease recurrence [3, 16, 20, 25, 44, 45]. Accidental introduction of the vector into or near houses may happen through movement of human belongings like clothes or blankets, movement of chickens carrying early instar nymphs or transportation of infested wood or palm leaves [16, 44]. In addition, local wildlife populations in peridomestic or sylvatic environments, such as bats, rodents and opossums, may serve as parasite reservoirs [20, 25, 46].

Given the diversity of interacting biotic elements involved in the epidemiology of Chagas disease, having simultaneous information on parasites, vectors, gut fauna and hosts would facilitate identifying how they interact to influence disease risk. Although genetic studies are typically focused on a single target organism at a time, reduced representation sequencing methods such as Restriction-site Associated DNA sequencing (RADseq) provide an affordable way to simultaneously sequence mixed-DNA specimens without relying on taxon-specific primers or probes [47]. When combined with a bioinformatics pipeline designed to identify and assign sequences back to their taxonomic source, such approaches may be ideally suited to explore complex, multi-factorial systems such as *T*. *cruzi* transmission cycles [48, 49]. RADseq also typically generates sufficient SNP loci to resolve relationships across multiple spatial and temporal scales, allowing a uniform protocol for producing data that can be meaningfully compared across studies [50, 51]. Although RADseq has been used to assess the population genomics of individual disease vectors (e.g., *Anopheles spp*., [52]; *Aedes aegypti*, [53]), it has not yet been reported for mixed-species analyses.

In this study, we develop a RADseq-based analysis pipeline for analyzing mixed-species DNA derived from *T*. *dimidiata* abdominal DNA. The ideal approach would be cost-effective, feasible with samples of varying age and quality, and capable of resolving vector and parasite population processes across spatial scales, from within-village dispersal to broad biogeographic and ecological differentiation. To evaluate whether the method was effective across this spatial range, we used a nested spatial sampling design for *T*. *dimidiata*, starting with multiple insects within and among individual villages, to samples collected from increasingly greater distances across major biogeographic regions in Central America. Sample results helped determine the utility of RADseq genotyping for simultaneous assessment of: (1) the prevalence of *T*. *cruzi* infection in the vector and its phylogenetic characterization in the region, (2) the population genetic structure of *T*. *dimidiata*, (3) the gut microbial community structure associated with *T*. *cruzi* infection of the vector, and (4) the blood meal history of the vector. We demonstrate that the method can effectively separate genomic information of parasite, vector, microbiome and blood meal, even without a sequenced genome for *T*. *dimidiata*.

## Methods

### Specimen collection, parasite screening and preservation

Sixty-one adult *T*. *dimidiata* were collected by the Laboratorio de Entomoligía Aplicada y Parasitología (LENAP) at San Carlos University of Guatemala and the Centro de Investigación y Desarrollo en Salud (CENSALUD) at Universidad de El Salvador from 1999 to 2013, representing a range of age and preservation conditions for evaluating the effect of specimen quality on sequencing yield (Table 1). Specimens were captured alive in domestic environments, transferred to a laboratory setting for microscopic examination for *T*. *cruzi* and placed in vials containing 95% ethanol + 5% glycerol within two days of capture. The exceptions were the specimens from the towns of El Chaperno and El Carrizal, collected in 2012 and 2013 (Table 1), which were examined by microscopy and placed in 95% ethanol (no glycerol) within a few hours of collection. To assess infection status, the abdomen of each insect was compressed to obtain fecal droplets that were diluted with 1 drop of saline solution and examined by a trained observer under the microscope at 220–400X for 5 minutes for active trypanosomes. The specimens placed in ethanol + glycerol were stored at room temperature at LENAP until being transported to Loyola University New Orleans or the University of Vermont in 2012 and 2013, respectively. Once in the United States, the insects were stored at -20°C until DNA was extracted for sequencing. Specimens from El Chaperno and El Carrizal were stored in ethanol at room temperature for less than one week before being transported to University of Vermont, where they were maintained at -20°C.

**Table 1:** Collection information for *Triatoma dimidiata* specimens used for RAD-sequencing.

ID	Body part extracted	Sex/Stage	Year	Lat	Long	Town	Municipality	Region	Country
**BLZ-01**	abdomen	Female	2008	16.2459	-88.8489	NA	Río Frío	Toledo	Belize
**SACH-01**	leg	Female	2009	14.0686	-89.5262	Chilcuyo	Santa Ana	Santa Ana	El Salvador
**SACH-02**	leg	Female	2009	14.0688	-89.5260	Chilcuyo	Santa Ana	Santa Ana	El Salvador
**SACH-03**	leg	Male	2009	14.0677	-89.5273	Chilcuyo	Santa Ana	Santa Ana	El Salvador
**SABE-01**	abdomen	Male	2009	14.1589	-89.4660	La Bedición	Santa Ana	Santa Ana	El Salvador
**SASA-01**	abdomen	Female	2009	13.9792	-89.5321	Santa Ana	Santa Ana	Santa Ana	El Salvador
**SASA-02**	leg	Female	2009	14.0002	-89.5150	Monte Largo	Santa Ana	Santa Ana	El Salvador
**SAJU-01**	leg	Male	2009	14.1152	-89.6424	El Jute	Texistepeque	Santa Ana	El Salvador
**SAJU-02**	leg	Female	2009	14.1150	-89.6409	El Jute	Texistepeque	Santa Ana	El Salvador
**CHAM-01**	leg	Female	2011	14.7411	-89.2395	Amatillo	Olopa	Chiquimula	Guatemala
**CHAM-02**	leg	Female	2011	14.7401	-89.2359	Amatillo	Olopa	Chiquimula	Guatemala
**CHCE-01**	abdomen	Female	2011	14.7097	-89.2865	El Cerrón	Olopa	Chiquimula	Guatemala
**CHCE-02**	abdomen	Male	2011	14.7359	-89.2397	El Cerrón	Olopa	Chiquimula	Guatemala
**CHCE-03**	leg	Female	2011	14.7119	-89.2849	El Cerron	Olopa	Chiquimula	Guatemala
**CHGU-01**	abdomen	Female	2011	14.7028	-89.3782	El Guayabo	Olopa	Chiquimula	Guatemala
**CHPR-01**	leg	Male	2011	14.7256	-89.2641	La Prensa	Olopa	Chiquimula	Guatemala
**CHPR-02**	abdomen	Female	2011	14.7214	-89.2718	La Prensa	Olopa	Chiquimula	Guatemala
**CHPR-03**	leg	Female	2011	14.7225	-89.2760	La Prensa	Olopa	Chiquimula	Guatemala
**JUCA-01**	abdomen	Male	1999	14.3741	-89.9844	El Carrizal	Jutiapa	Jutiapa	Guatemala
**JUCA-02a**	abdomen	Male	2013	14.3767	-89.9920	El Carrizal	Jutiapa	Jutiapa	Guatemala
**JUCA-02b**	leg	Male	2013	14.3767	-89.9920	El Carrizal	Jutiapa	Jutiapa	Guatemala
**JUCA-03**	abdomen	3rd stage nymph	2013	14.3720	-89.9836	El Carrizal	Jutiapa	Jutiapa	Guatemala
**JUCH-01**	abdomen	Female	2012	14.3473	-89.9483	El Chaperno	Jutiapa	Jutiapa	Guatemala
**JUCH-02**	abdomen	Male	2012	14.3434	-89.9446	El Chaperno	Jutiapa	Jutiapa	Guatemala
**JUCH-03**	abdomen	Male	2012	14.3621	-89.9476	El Chaperno	Jutiapa	Jutiapa	Guatemala
**JUCH-04**	abdomen	Female	2012	14.3523	-89.9456	El Chaperno	Jutiapa	Jutiapa	Guatemala
**JUBR-01**	abdomen	Female	2012	14.3289	-90.0625	La Brea	Jutiapa	Jutiapa	Guatemala
**UnID**	abdomen	Unknown	NA	NA	NA	NA	NA	NA	Guatemala
**PTN-01**	abdomen	Female	2012	16.6932	-89.4390	Chapayal	San Luís	Petén	Guatemala
**PTN-02**	abdomen	Male	2012	15.4970	-90.9818	Chapayal	San Luís	Petén	Guatemala
**QUI-01**	abdomen	Male	2004	15.4970	-90.9818	Tzitzima	San Andrés Sajcabajaj	Quiché	Guatemala
**NIC-01**	abdomen	Unknown	2007	13.4656	-86.4588	San Ramón	Palacaguina	Madriz	Nicaragua

**Fig 1:**
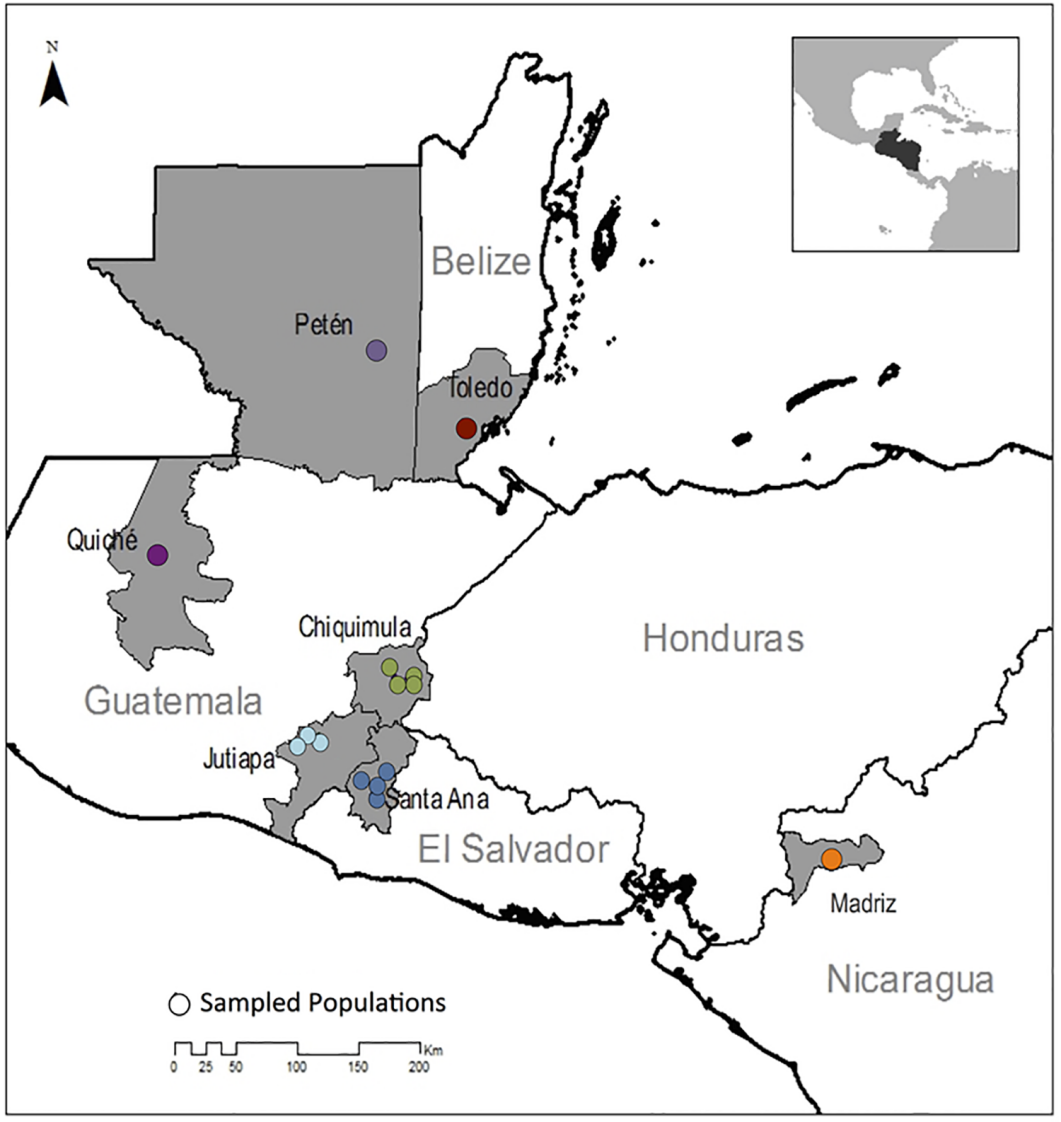
Geographic locations of the sequenced *T*. *dimidiata* specimens. Specimens from Madriz, Nicaragua, Quiché, Guatemala, Petén, Guatemala and Toledo, Belize were sampled to capture variation across countries. To assess within-country regional diversity, specimens from Guatemala and El Salvador were sampled more intensively to include regional and village-scale variation. Locations are color-coded by the Within-Country Regions.

To measure the spatial resolution at which RADseq markers are able to resolve the genetic structure of *T*. *dimidiata* and *T*. *cruzi*, three nested geographical spatial scales of sampling were selected: a) individual villages, including five in the neighboring regions of Chiquimula, Jutiapa, and Santa Ana; b) within-country regions, including three in Guatemala, and one in El Salvador; and c) countries across Central America, including Guatemala, Belize, El Salvador and Nicaragua (Table 1, Fig 1).

### DNA extraction and RAD-library preparation

We extracted DNA from the 61 specimens from the three posterior segments of the abdomen or four surface-sterilized legs (Table 1); the latter included the attached muscle, and served as “insect-only” controls. Tissues were flash-frozen by submerging the vials in liquid nitrogen, manually homogenized using sterilized pestles and DNA extracted using a modified Qiagen DNeasy (Burlington, Vermont) tissue extraction protocol. Modifications included an overnight Proteinase K digestion at 56°C, followed by an RNAse digestion at 37°C for 30 minutes using 1.5 uL of 4mg/mL RNAse to reduce RNA contamination. DNA was quantified using a Qubit spectrophotometer (Burlington, Vermont), and quality was assessed by electrophoresis on a 1.5% agarose gel stained with ethidium bromide. Only specimens with a minimum yield of 1,000 ng of DNA and a single, high-molecular weight band were considered suitable for sequencing; of the original 61 specimens, 32 (20 abdomens and 12 legs) met these minimal requirements. To verify the reproducibility of the retrieved genetic markers (SNPs), for one insect specimen we included high-quality DNA isolated from two different body parts (abdomen and leg tissue, JUCA-02A and JUCA-02L; Table 1). RADseq library preparation was conducted using the restriction enzyme *SbfI* (8-base cutter: 5′—CCTGCA↓GG—3′, 3′—GG↓ACGTCC—5′) at Floragenex (Portland, Oregon) following the methods of Baird et al. [47].

### Illumina sequencing and bioinformatics pipeline

RAD libraries were barcoded by individual, and multiplexed in a 24-plex format on an Illumina GAIIx / HiSeq Analyzer. The raw sequencing reads were 100 bp in length, including the inline 5-bp barcode and 8-base*SbfI* recognition sequences. We used FastX-trimmer in the FastX-toolkit to remove the barcodes, recognition sites, and FastQ-quality-filter to remove sequences with any base having a confidence score below 10 [54].

**Fig 2:**
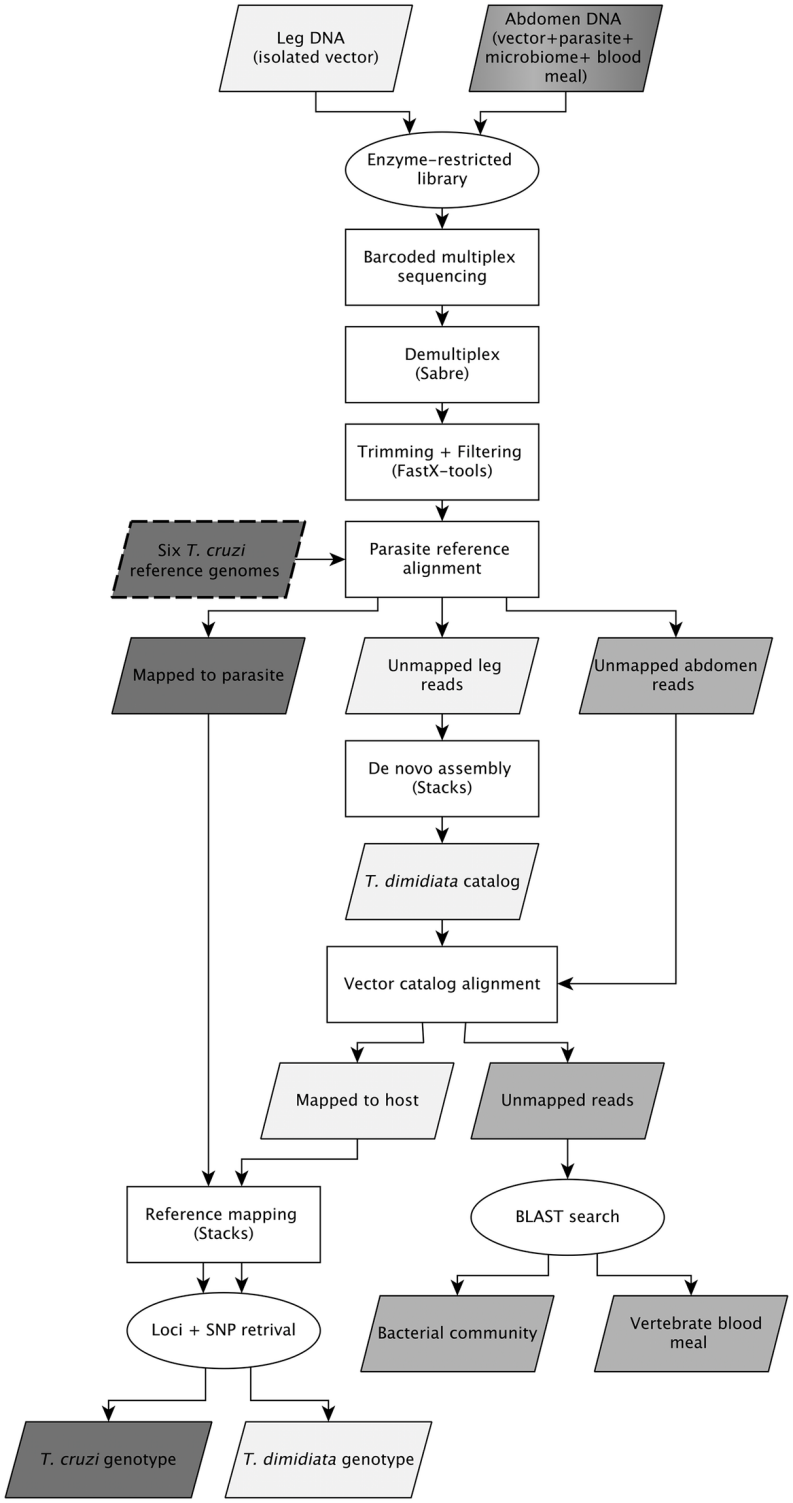
Bioinformatics pipeline separating RADseq data obtained from the legs and abdomens of *Triatoma dimidiata* specimens. Raw data from 32 *T*. *dimidiata* were trimmed and filtered using FastX tools, then mapped to the six available *T*. *cruzi* genomes using Bowtie. The unmapped reads from the host were assembled *denovo* using Stacks, converted to an index, and used as a catalog to map all to *T*. *dimidiata*; both sets of mapped reads were aligned in STACKS to obtain markers for the parasite and host. The NCBI nt database was queried (May, 2016) with the remaining unmapped reads to quantify matches obtained from chordates (blood meal hosts), bacteria and other taxa. Input and output parallelograms are color-coded to indicate the vector (yellow), parasite (pink) and all other taxa (orange).

The DNA recovered from a *T*. *dimidiata* abdomen represents a mixture of DNA from the parasite (if present), the insect vector, possibly one or more vertebrate blood meals, and the microbial community residing in the gut, internal tissues and on the cuticle. We designed a custom bioinformatics pipeline to separate these DNA sources and analyzed them individually for either SNP genotypes (*T*. *dimidiata*, *T*. *cruzi*) or taxonomic identification (blood meal, microbes) (Fig 2).

We mapped the trimmed sequences from all 32 specimens against six *T*. *cruzi* reference genomes downloaded from the NCBI genome database (May, 2016) using Bowtie 1.1.2 [55]. These included a subset of DTUs: two representatives of TcI (ACCN: AODP01000000, ADWP02000000), one of TcII (ACCN: ANOX01000000), and two of TcVI (ACCN: AAHK01000000, AQHO01000000). We also included *T*. *cruzi marinkellei* (ACCN: AHKC01000000), which served as the phylogenetic out-group. The 12 samples of *T*. *dimidiata* leg tissue were also mapped to the *T*. *cruzi* genomes in order to filter out any possible *T*. *cruzi* contamination from handling, with only the unmapped reads from this step used in downstream analyses. Mapping success was negligible (< 8 reads) for all of the leg samples.

Because there is no sequenced genome for *T*. *dimidiata*, we used the sequences derived from leg tissue to assemble a reference set of RAD-tags most likely to be derived from the *T*. *dimidiata* genome. Using the 12 legs, we used the denovo_map pipeline in Stacks to obtain a putative set of *T*. *dimidiata* loci [56] (Fig 2). The parameters of the alignment were set at 3X depth of coverage for the initial stack, with a maximum of two mismatches among trimmed sequences of a single individual. Once the first stack was formed with primary reads that met the parameters, we allowed a maximum of 4 mismatches when aligning the secondary reads (those reads that did not meet the cut-off to align in the first stack), and a maximum of 3 mismatches per nucleotide across both the primary and secondary reads [56]. Once the alignment yielded a raw catalog, tags were retained if: (a) at least half of the specimens had a read for the locus, (b) there were between 0 and 3 SNPs present across the reference sequences and (c) there were no more than two haplotypes for any individual specimen at the locus. A total of 6206 loci fitting these criteria were used as a custom index in Bowtie against which all 32 specimens were mapped to obtain individual, vector-specific reads (Fig 2).

SNP genotypes for both *T*. *cruzi* and *T*. *dimidiata* were called using the Stacks ref_map pipeline [56]. Because the number of reads retrieved for the vector were an order of magnitude higher than for the parasite (see Results), we set the parameters for the vector to a maximum of six mismatches between loci and a depth of coverage of 3X, while for the parasite we also allowed up to 6 mismatches but retained calls at 1X depth of coverage. We excluded any locus with missing data in at least 18 of the 32 specimens for *T*. *dimidiata* and 10 of the 13 *T*. *cruzi*-positive specimens for *T*. *cruzi*.

With the remaining unmapped reads, we ran a BLAST search query of the nt database for potential blood-meal sources and microbiota, using an e-value cutoff of 0.001, a query coverage minimum of 85 bp (97%), and only retaining the top hit that mapped to each sequence (Fig 2). Exploratory mapping to other databases (e.g., RefSeq) yielded fewer hits than the nt database and were not included in the final pipeline. When the sequence mapped equally well to multiple taxa, the first species returned by the BLAST algorithm was retained; although species identity in such cases was not well supported, identification was consistent across all reads of identical sequence within and among specimens. Information on the mean e-value cutoffs by taxonomic group is provided as S1 Table.

### Data analysis

Because genomic reference sequences were available for only three of the six DTUs, two approaches were used to assign a putative DTU to the *T*. *cruzi*-positive specimens. First, we identified the total set of reads for each specimen that mapped successfully to any one or more of the *T*. *cruzi* reference genomes and then mapped this set of reads to each genome individually to determine relative mapping success. For comparison, we generated *in-silico* RAD-tags from the six reference genomes using a custom python script that identified all occurrences of the restriction enzyme recognition sequence in the genome and retrieved the 87 bp directly up- and down-stream of the cut site. These were mapped against each of the six reference genomes using the same Bowtie protocol as with the field specimen data to obtain expected mapping success for a given DTU. Two main patterns of mapping success were found across the entire DNA specimen set (see Results); for each distinct subset, we ran one-way ANOVA and a post-hoc Tukey’s range test using the *stats* package in R [57] to test whether the mapping success was biased toward a particular reference genome. Second, we used the SNP genotypes generated with Stacks to reconstruct phylogenetic relationships among the *in-silico* genomes and the field specimens with MEGA version 7, using Maximum Likelihood with a nucleotide p-distance substitution model and 10,000 bootstrap permutations [58].

To infer the population genetic structure of *T*. *dimidiata*, we performed a k-means clustering analysis, and classified the individuals by a discriminant analysis of principal components (DAPC) using the *Adegenet* package for R [59]. To prevent biases associated with missing data, specimens with >50% missing SNPs were excluded from the analysis (i.e., CHGU-01 and CHCE-01); one additional specimen (UnID) did not have precise geo-location information and was also excluded. Using the 29 remaining specimens, we identified the best number of genetic clusters using the k-means cluster algorithm from the find.clusters function in *Adegenet* and selected the value of k that minimized the Bayesian Information Criterion (BIC) value, setting the maximum number of potential clusters to 16, and retaining a total of 25 principal components based on the cumulative variance explained by the eigenvalues. We also calculated the fixation index (Fst), nucleotide diversity (pi), observed (Het_ob_) and expected (Het_ex_) heterozygosity among clusters using the *Populations* function in Stacks [56].

To compare bacterial species richness across specimen types (infected abdomens, non-infected abdomens and legs), we used the rarefaction function in the *Vegan* package in R to estimate asymptotic species richness for each specimen [60,61]. Specimen types were compared using an ANOVA with post-hoc Tukey’s pairwise comparisons in the R *Stats* package. To compare gut bacterial community composition as a function of infection status, we ran a non-metric multidimensional scaling (NMDS) weighted Unifrac ordination analysis with the default number of dimensions (k = 2) using the *phyloseq* package in R [62]. Because they do not contain gut tissue, leg specimens were excluded from this analysis. Bacterial phylogenetic relationships were retrieved from the SILVA 123 ribosomal living tree, pruned to the set of taxa present in the specimens using the *prunedTree* function in the *Picante* package [63, 64 65]. The matrix of counts is available in S3 Table. To assess significance of clusters, we performed a post-hoc permutation analysis of 999 repetitions embedded in the NMDS function.

To distinguish actual vertebrate blood meals from possible contamination due to handling and/or false-positive BLAST hits from multiple taxon matches, we identified the chordate species identified by the largest number of sequencing reads (the "top-hit" species) for each specimen. Representation of the top-hit species within a specimen was expressed as a percent of the total possible hits (i.e., the total number of reads that had not mapped to either the parasite or vector). Leg specimens were used to determine the expected background representation of chordate hits. Putative blood meals were called for those specimens with a top-hit representation statistically above the background, identified with an outlier test using the Tukey boxplot method for skewed data [66], with the upper outlier threshold defined by the Tukey range of Q_3_+1.5*IQR, the Inter-Quartile Range (S4 Table).

## Results

### Using RADseq for multi-organism mapping

We obtained a total of 164.1 million unfiltered reads across all specimens. There was no difference in the number of raw reads between leg and abdomen, or among specimens obtained in different collection years. After quality filtering, 70.69% of reads were retained, with an average of 3.25 million reads per specimen (± 652,000).

**Fig 3:**
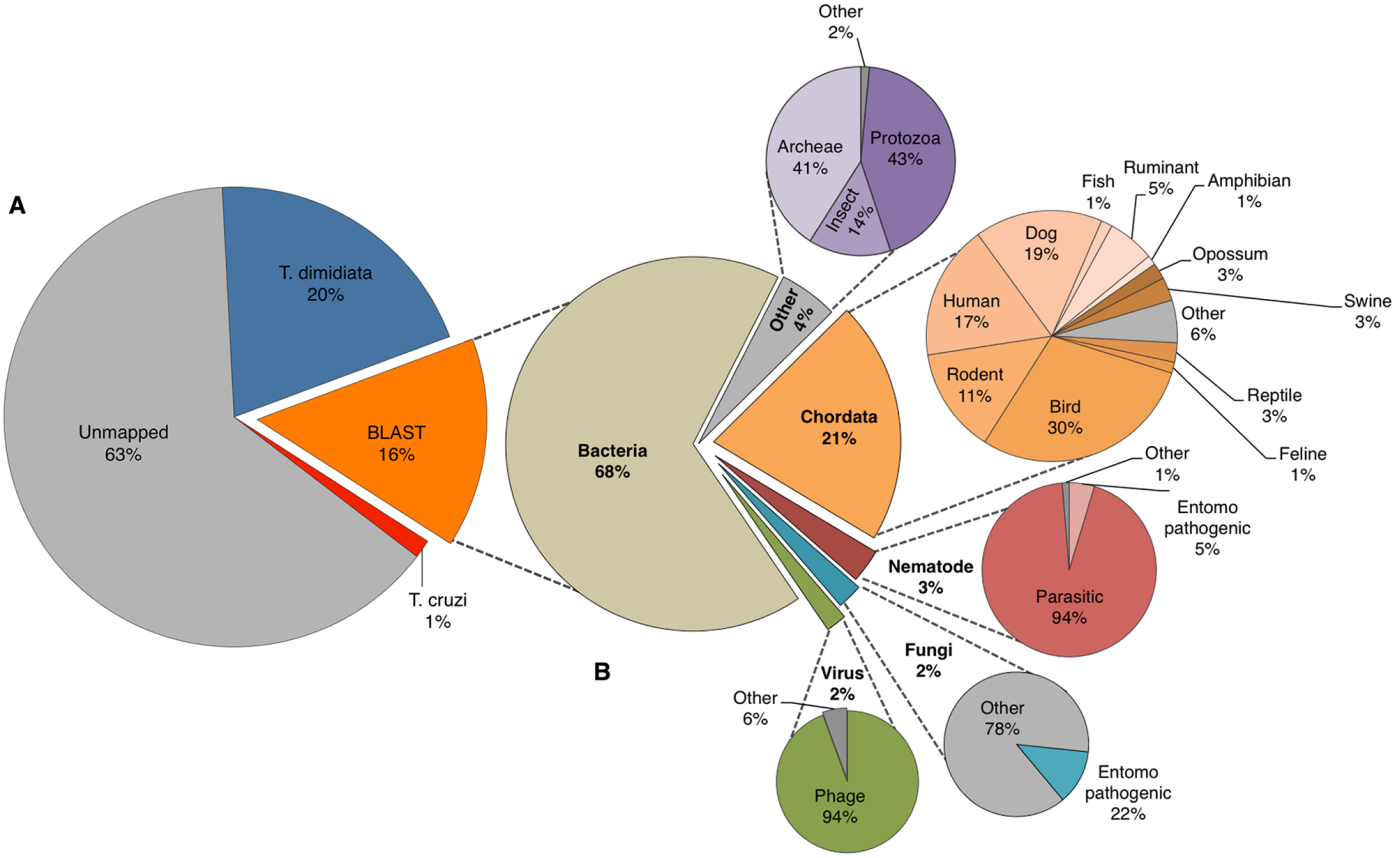
Percentage of reads mapped to different DNA sources across all specimens. (A) The overall percentage of reads mapped to *Trypanosoma cruzi*, *Triatoma dimidiata*, other taxa (BLAST results), and unmapped reads; and (B) the breakdown of taxa retrieved from a BLAST search using the nt database from NCBI.

**Fig 4:**
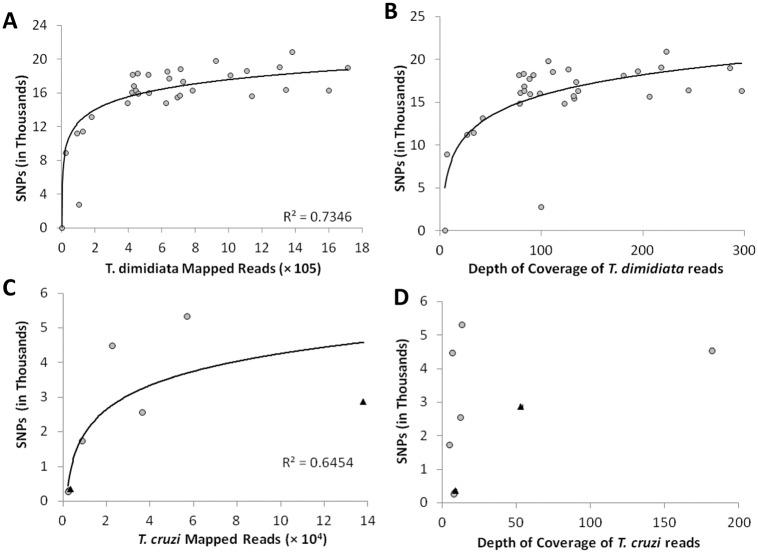
Number of SNPs retrieved in relation to mapped reads and depth of coverage for *T*. *dimidiata* and *T*. *cruzi*. Log-transformed number of single nucleotide polymorphisms (SNPs) in relation to the number of (A) *T*. *dimidiata* and (B) *T*. *cruzi* mapped reads, and the average depth of coverage for (C) *T*. *dimidiata* and (D) *T*. *cruzi*. In panels B and D, gray circles indicate putative TcI and black triangles indicate putative TcIV specimens.

Analysis with the mixed-species pipeline produced subsets of reads corresponding to all of the expected taxonomic groups (parasite, vector, blood meal and bacteria) (Fig 3). Although the majority of reads (63%) could not be assigned to a particular source, both the vector (20%) and the parasite when present (1%) were represented by sufficient mapped reads to approach saturation of SNP recovery (Figs 3a, 4a and 4b). In our internal control (Table 1), the leg specimen (JUCA-03) was over-represented compared to the abdomen (JUCA-02) from the same insect, yielding 60.8% more trimmed reads than the abdomen. This difference affected the number of mapped reads (37.87% higher), mean depth of coverage (222.8X for leg versus 98X for abdomen; Fig 4c), and number of called SNPs (19% higher); however, for the 15,611 loci called across both genotypes, only eight (0.05%) were different between the two tissue types.

### *Trypanosoma cruzi* infection and phylogenetic identification of parasite DTUs

**Fig 5:**
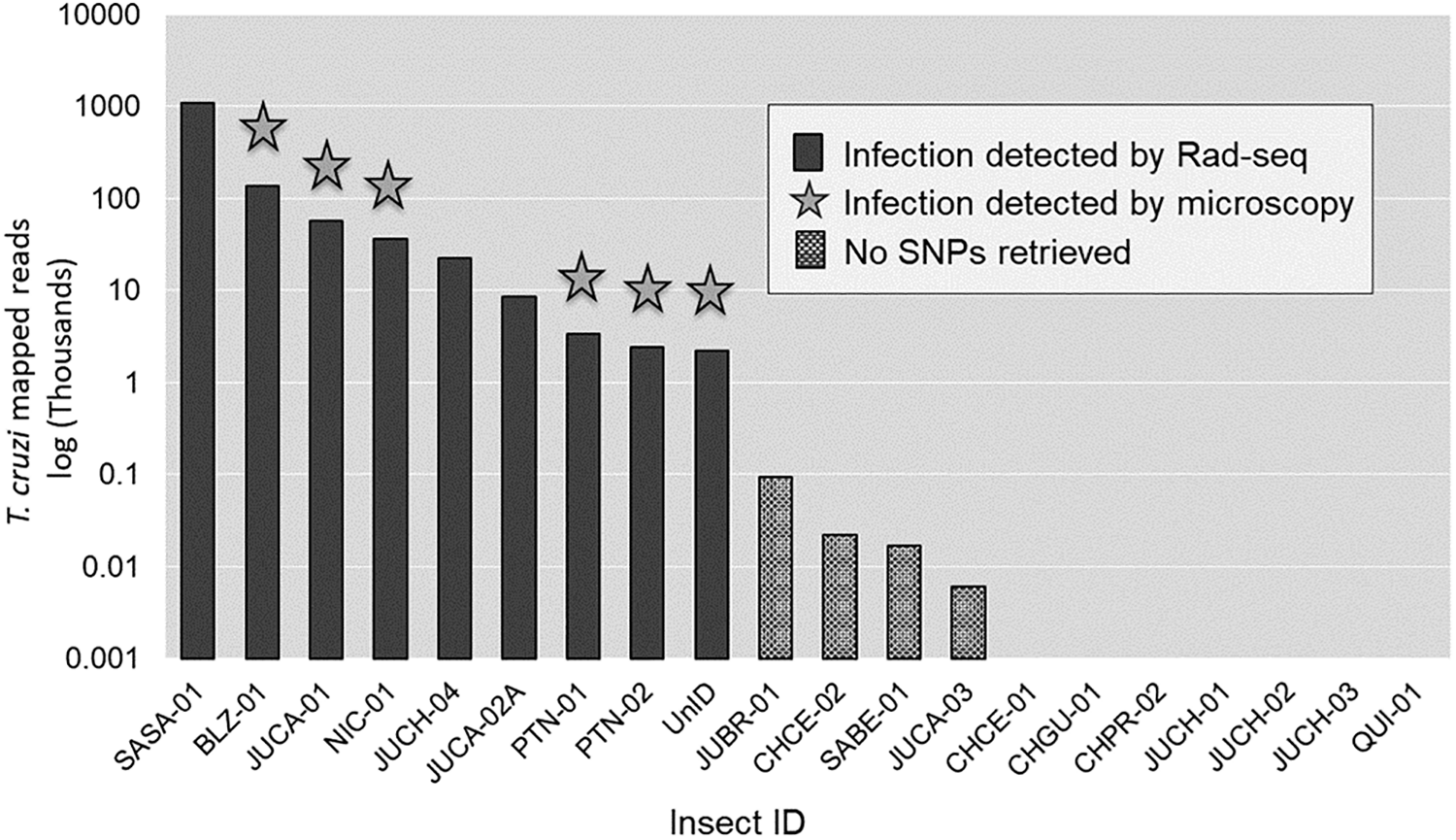
*Trypanosoma cruzi* infection measured by the count of mapped reads detected from the 20 genotyped abdomens. Star indicates positive *T*. *cruzi* infection detected by microscopy. CHCE -01 through QUI-01 have zero mapped reads.

Thirteen of the 20 abdomens mapped to at least one of the six available *T*. *cruzi* reference genomes; however, four of these specimens yielded fewer than 100 mapped reads, with no polymorphic loci (Fig 5). These specimens were omitted from further *T*. *cruzi* analysis. Eight of the 12 leg specimens did not map to any of the *T*. *cruzi* genomes, while four legs mapped to at least one genome with a range of 1–7 reads and no polymorphic loci. The nine *T*. *cruzi*-positive abdomens yielded an average of 150,994 ±118,089 mapped reads, corresponding to 6,377 unique genomic locations, with a total of 6,405 SNPs (Fig 5). The median depth of coverage was 8.7X, ranging from 4.7X to 181.9X; there was no relationship between the mean depth of coverage and the number of SNP genotypes successfully called per specimen (Fig 4D).

Detection of infection status via fecal microscopy and RADseq were significantly associated (Fisher’s Exact test, p = 0.0018) (Fig 5). All six specimens positive for *T*. *cruzi* by microscopy were also positive by RADseq. Seven additional *T*. *cruzi*-positive specimens were detected by RADseq but not by microscopy, including three with high read abundance and the four that yielded <100 reads. Among the positive specimens identified solely by this method, the abdomen internal control, JUCA-02A, yielded a total of 8,610 *T*. *cruzi* reads. In contrast, the leg control extracted from the same insect, JUCA-02L, yielded only 7 *T*. *cruzi* reads.

Genome mapping comparisons indicated that the nine *T*. *cruzi* isolates from the *T*. *dimidiata* abdomens included two distinct parasite DTUs (Table 2). Patterns of mapping success fell into two distinct groups; one encompassed the geographical range from Petén to Nicaragua (i.e. JUCA-01, PTN-01, PTN-02, NIC-01, JUCA-02, JUCH-04, SASA-01), while a second group included Belize (BLZ-01) and an unidentified specimen from Guatemala (UnID) (Table 2). Specimens from the first group were most similar to the TcI DTU (>92% mapping success to TcI-AODP, >74% TcI-ADWP), followed by TcVI (<64%), TcII (<46%) and *T*. *c*. *marinkellei* (<12%) (Table 2). This was consistent with the TcI *in-silico* specimen, which mapped more successfully to the TcI reference genome than to any other DTU. Specimens from the second group mapped most closely to TcVI, consistently mapping >91% of their reads to the two available TcVI genomes, followed by TcII (<76%), TcI (<70%) and *T*. *c*. *marinkellei* (<12%), respectively (Table 2). This pattern was most similar to the TcVI *in-silico* reads, although compared to the TcVI *in-silico* tags, mapping success of the field specimens was lower for the TcVI genomes and higher for TcI and TcII (Table 2).

**Table 2:** Percentage of reads independently mapped to six *T*. *cruzi* reference genomes.

Type	DTU* / Specimen ID	TcI		TcII	TcVI		*Tc marinkellei*
		AODP	ADWP	ANOX	AAHK	AQHO	AHKC
Reference Genomes (%)	TcI—AODP	100	81	39	56	54	8
	TcI—ADWP	84	100	41	60	58	7
	TcII—ANOX	41	41	100	85	84	10
	TcVI—AAHK	46	45	63	100	94	10
	TcVI—AQHO	46	44	62	96	100	10
	*Tc marinkellei*—AHKC	8	6	1	0	12	100
Field Specimens (%)	JUCA-01	93	79	42	58	56	9
	PTN-01	95	86	46	62	62	9
	PTN-02	95	84	44	64	60	12
	NIC-01	96	87	44	62	60	7
	JUCA-02	95	74	37	52	50	7
	JUCH-04	92	83	44	61	58	10
	SASA-01	96	86	44	62	60	8
	**Tukey’s range test**	**a**	**b**	**d**	**c**	**c**	**e**
	BLZ-01	70	69	70	92	91	11
	UnID	64	64	76	92	91	12
	**Tukey’s range test**	**b c**	**c**	**b**	**a**	**a**	**d**

*DTU accession numbers = AODP00000000.1, ADWP00000000.2, ANOX00000000.1, AAHK00000000.1, AQHO00000000.1, and AHKC00000000.1. Letters a-e corresponds to the separation of means determined by a post-hoc Tukey’s range test (p>0.001).

**Fig 6:**
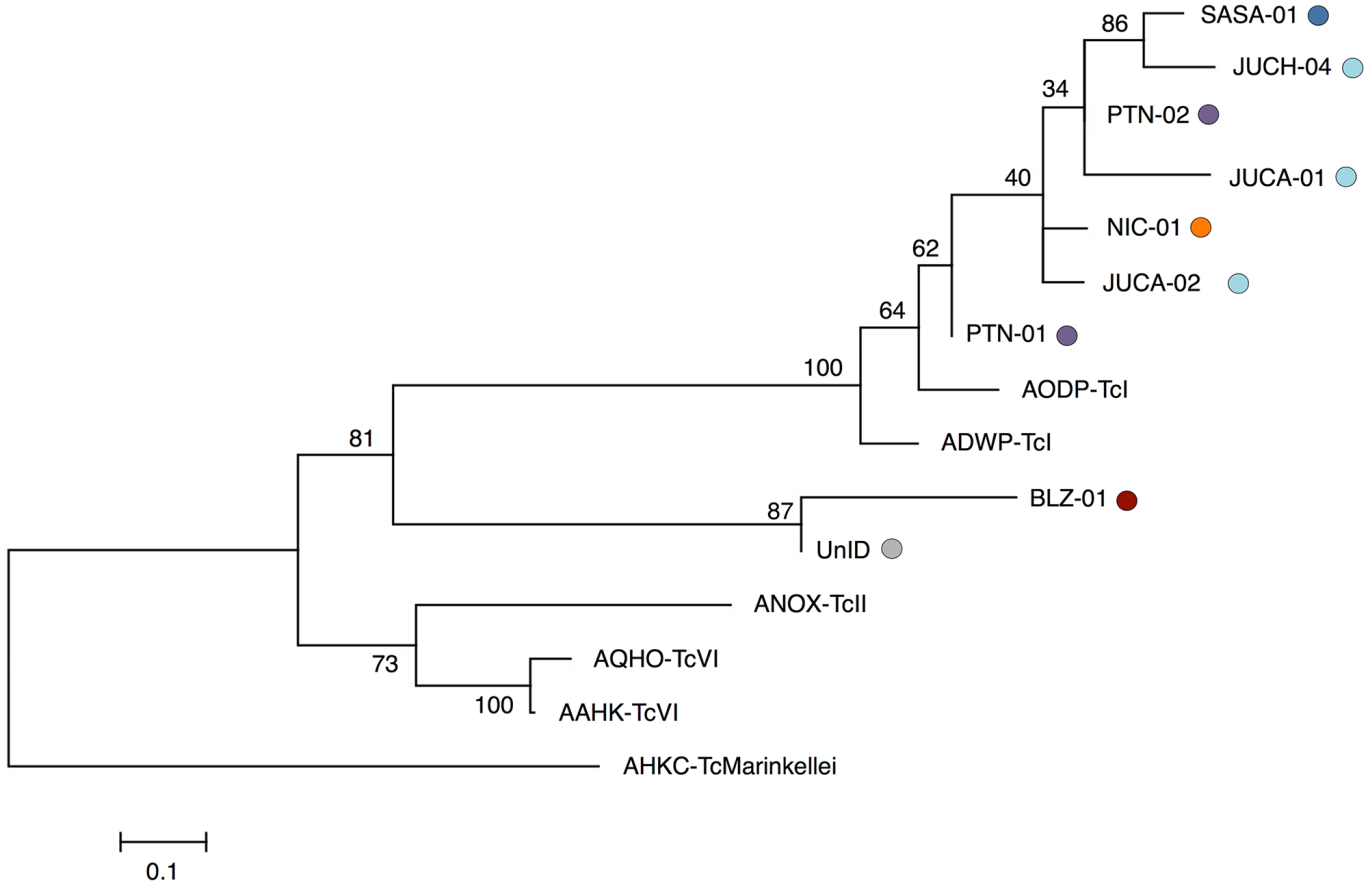
Phylogenetic inference by maximum likelihood of *T*. *cruzi* from nine infected abdomens of *T*. *dimidiata*. Specimens originating from Petén, Guatemala (Purple) Jutiapa, Guatemala (light-blue), Belize (red), Santa Ana, El Salvador (blue) and Nicaragua (orange), and six *in-silico* genotypes from the reference genomes of two TcI, one TcII, and two TcVI DTUs and the out-group *T*. *c*. *marinkellei*. The tree topology was tested with 10,000 bootstrap replications, using a total of 34,707 bi-allelic SNPs.

Phylogenetic reconstruction also supported the existence of two DTUs (Fig 6). Although most specimens clustered with strong bootstrap support into a single clade with the two TcI genome references, the exceptions were BLZ-01 and UnID, which formed a distinct cluster, sister to TcI and distinct from the clade that includes the TcVI and TcII reference genomes (Fig 6).

### Genetic variation in *Triatoma dimidiata*

All leg and abdomen samples mapped successfully to the *T*. *dimidiata* reference catalog, with an average of 610,013 ± 80,410 mapped reads, corresponding to 19,577 ± 4,389 tags, and a total of 25,710 *T*. *dimidiata* SNPs across the 32 specimens. Of these, individual villages contained from 9–27% of the total allelic variation, resulting in over 1900 informative SNPs even at the smallest spatial scale assayed (Table 3). As the scale was increased from villages to regions, polymorphism was detected at an increasing proportion of SNPs, with the region of Jutiapa containing nearly 50% of the total number identified across the entire area of the study.

**Table 3:** Number and proportion of SNPs recovered at each spatial scale. To account for missing data, the proportion of variable markers present in each subset was calculated from the total number of loci with sufficient data for analysis.

Town	Region	Country	No. samples	No. SNPs	No. Loci	Prop. Variable
***Within village***:						
El Carrizal	Jutiapa	Guatemala	3	5055	23674	0.21
El Chaperno	Jutiapa	Guatemala	4	6414	24096	0.27
La Prensa	Chiquimula	Guatemala	3	2513	23891	0.11
El Cerron	Chiquimula	Guatemala	3	1939	22217	0.09
Chilcuyo	Santa Ana	El Salvador	3	2237	23680	0.09
***Within region***:						
	Jutiapa	Guatemala	8	12509	25491	0.49
	Chiquimula	Guatemala	9	5029	25666	0.20
	Santa Ana	El Salvador	8	5193	25611	0.20
	Peten	Guatemala	2	1995	19193	0.10
***Across regions (all samples)***:			32	25710	25710	1.00

**Fig 7:**
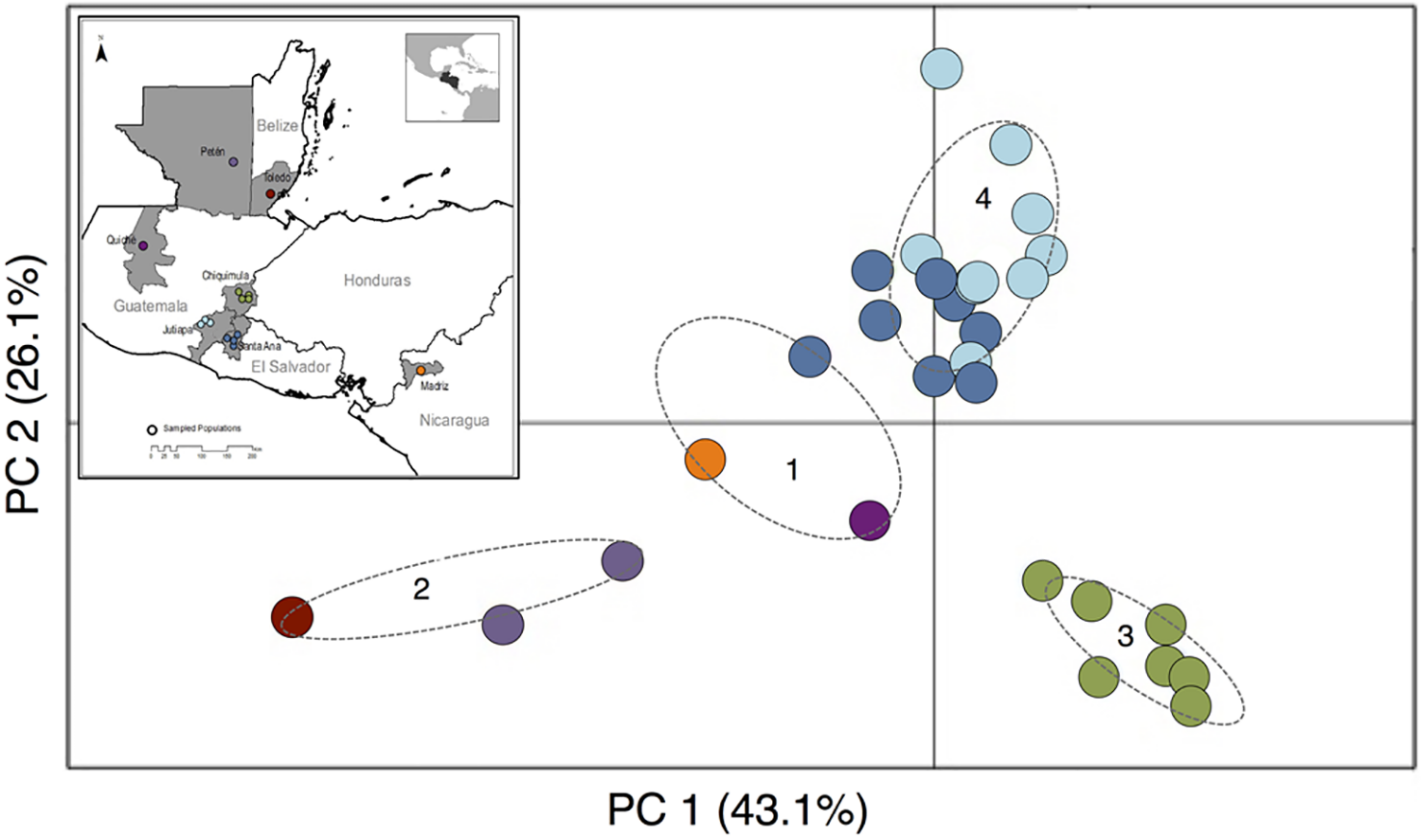
Population genetic structure of *Triatoma dimidiata* across Central America inferred with a discriminant analysis of principle components (DAPC) based on SNP markers. DAPC shows the maximized differences among four genetic clusters of the vector. Clusters were determined using the k-mean clustering algorithm and choosing the lowest Bayesian Information Criterion (BIC). Ellipses show 95% confidence intervals. The first two eigenvalues explain 69.2% of the variation found in 21,461 SNPs.

K-means clustering and posterior DAPC revealed four main clusters corresponding to their geographical distributions among the 29 *T*. *dimidiata* individuals included in the analysis (two excluded for low SNP counts, and one for which location data were not available) (Fig 7). Madriz, Nicaragua (NIC), Quiché, Guatemala (QUI) and La Bendición, El Salvador (SABE) were clustered in one group; the two northern sites, Río Frío, Belize (BLZ) and Petén, Guatemala (PTN), were clustered in a second group; all individuals from Chiquimula, Guatemala (CHAM, CHCE, CHGU and CHPR) were isolated in a third cluster; and the remaining specimens from the region of Santa Ana, El Salvador and Jutiapa, Guatemala (SACH, SAJU, SASA, JUBR, JUCA, JUCH and JUYU) were grouped in a fourth cluster (Fig 7). The *F*_*st*_ values between clusters were greater than zero in all pair-wise comparisons; cluster 3, which groups all individuals from Chiquimula, was the most differentiated, with pair-wise F_st_ ranging from 0.142 to 0.222 compared to 0.062 to 0.083 for all pair-wise combinations not involving cluster 3 (Table 4). Nucleotide diversity and observed heterozygosity were highest in cluster 4 (El Salvador + Jutiapa) compared to other clusters, despite the relatively small geographic area encompassed by this cluster (Table 4; Fig 1). Across all clusters, the expected heterozygosity tended to be higher than the observed (Table 4).

**Table 4:** F-statistics and summary statistics for *Triatoma dimidiata* clusters identified by k-means clustering.

*F*_*st*_	1	2	3	4	pi	Het_ex_	Het_ob_
1	0	0.063	0.142	0.062	0.086	0.068	0.045
2		0	0.222	0.083	0.099	0.078	0.056
3			0	0.165	0.093	0.084	0.041
4				0	0.170	0.159	0.102

Cluster 1 includes Nicaragua (NIC), Quiché (QUI) and La Bendición (SABE); cluster 2 includes Belize (BLZ) and Petén (PTN); cluster 3 includes Chiquimula (CHAM, CHCE, CHGU and CHPR); and cluster 4 includes Santa Ana and Jutiapa (SACH, SAJU, SASA, JUBR, JUCA, JUCH and JUYU). Cluster pair-wise F-statistics are color-coded by genetic differentiation, where darker grey shows stronger cluster differentiation. The individual pairwise distance values are available in S5 Table.

### Other taxa from BLAST search

For the 16% of reads with a significant BLAST hit (e-value < 0.001), 68% mapped to bacteria, 21% mapped to chordates, and the remaining 11% mapped to archaea, insects, protozoa, viruses, fungi and nematodes (Fig 3b). Among chordates, 59% matched to known mammalian *T*. *cruzi* hosts, including dogs, humans, rodents, cats, swine, ruminants and opossum (Fig 3b). Domestic birds, including chickens, ducks, and turkeys, constituted 30% of the bird BLAST reads. Within the viruses, 94% were bacteriophages. Fungal hits included entomopathogenic strains in the orders Hypocreales (e.g., *Beauveria* and *Metarhizium*) and Entomophthorales (e.g., *Zoophthora* and *Entomophaga*) typically used for biological control. Human and rodent parasitic nematodes, in the genera *Angiostrongylus*, *Heligmosomoides*, *Haemonchus*, *Parastrongyloides*, and *Strongyloides* constituted 94% of the nematode community and were found across all 32 specimens, while entomopathogenic nematodes from the genus *Steinernema* constituted 5% of the nematode mapped reads (Fig 3b).

### Gut bacterial community structure

**Fig 8:**
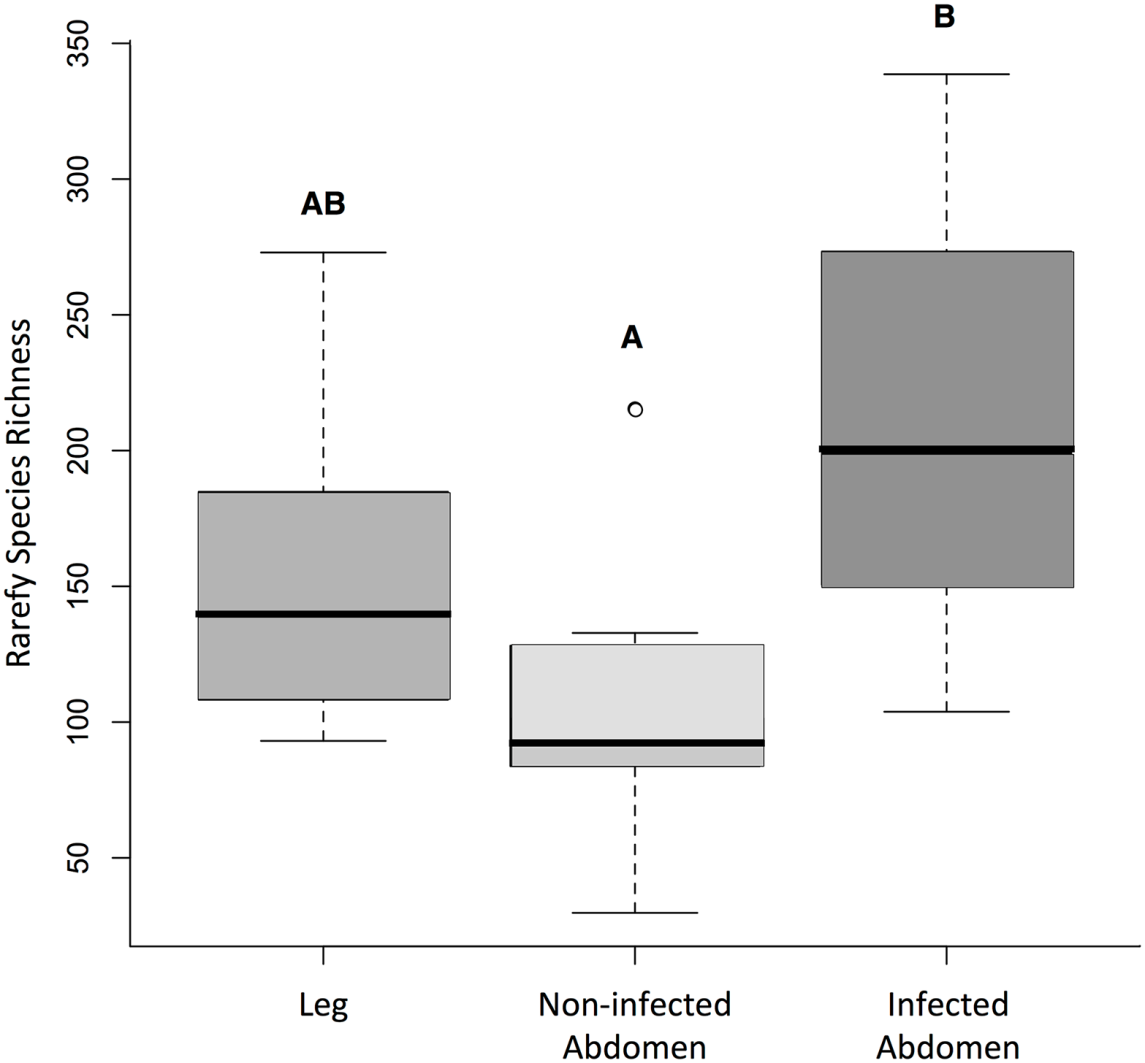
Box-plot comparison of the asymptotic species richness of identified in SNPs from *T*. *dimidiata* legs, non-infected abdomens and *T*. *cruzi*-infected abdomens. Letters indicate statistically significant groupings based on post-hoc Tukey’s tests (p <0.01).

Bacterial species richness varied significantly across specimen types (F_2, 29_ = 4.15, p = 0.019). Infected abdomens with *T*. *cruzi* contained significantly more bacterial species than non-infected abdomens (post-hoc Tukey test, p <0.01) (Fig 8), but there was no difference in species richness between the leg specimens and either infected or non-infected abdomens. We identified 1,142 putative bacterial species across all abdomens. The reads from the subset of *T*. *cruzi*-infected abdomens mapped to 1,006 bacterial species, with 49% unique to a single specimen and 28% present across more than 50% of the infected abdomens. SNPs from non-infected abdomens mapped to 508 bacterial species, with 70% of the species mapping to a single specimen; however, only 12 species (2.4%) from four genera (*Bacillus*, *Enterobacter*, *Ralstonia*, and *Alcaligenes*) were shared by more than 50% of the uninfected specimens.

**Fig 9:**
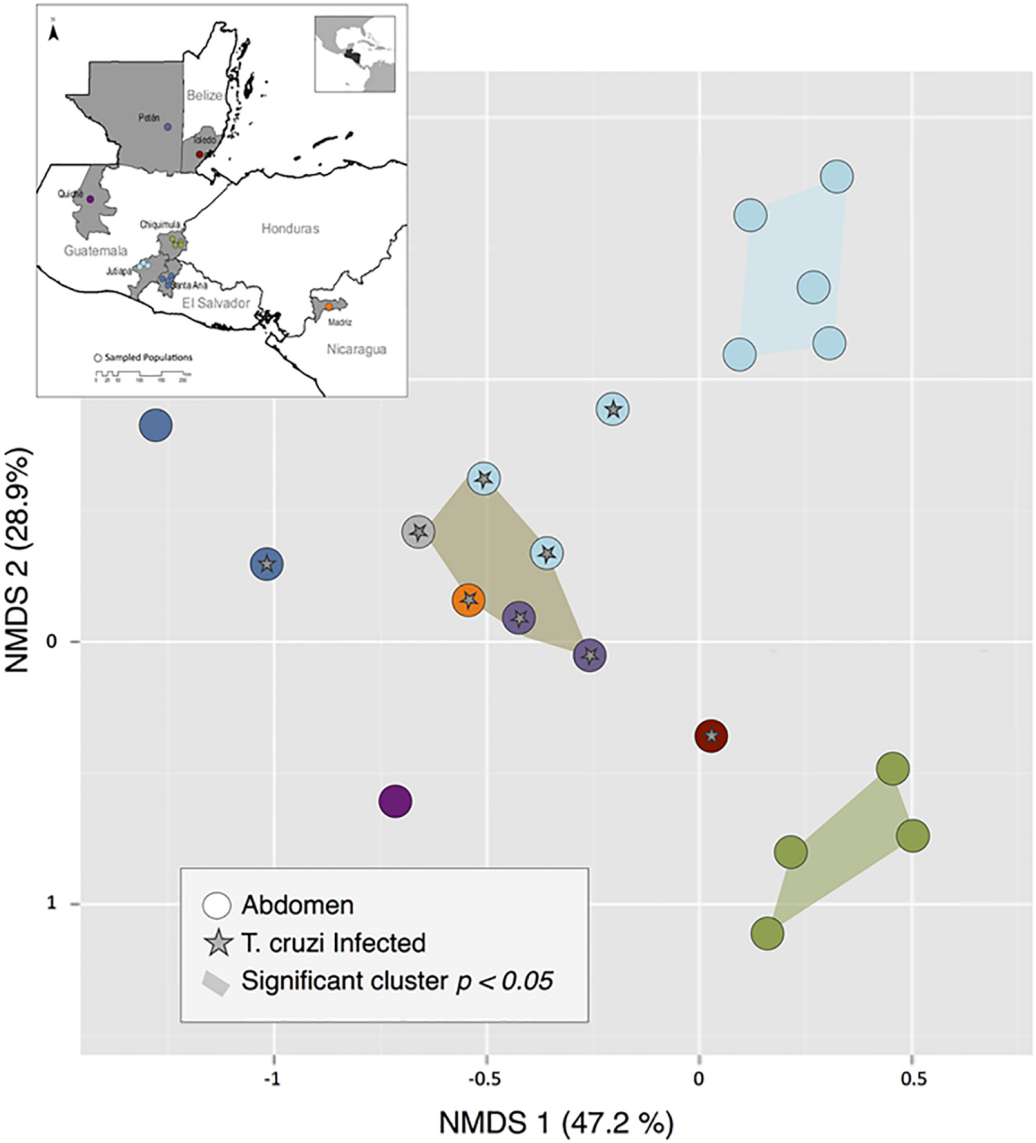
NMDS plot of bacterial community structure based on weighted Unifrac distances. Specimens are color-coded by within-country regions; stars indicate *T*. *cruzi*-positive abdomens. Colored polygons indicate statistically significant clusters from a post-hoc permutation test.

Unifrac analysis of gut bacterial community composition grouped specimens based on both geographic location and infection status. The first NMDS axis, explaining 47.2% of the variance, separated most regions from Guatemala and Belize from Quiché, Guatemala and El Salvador. The second NMDS axis, explaining 28.9% of the variance, separated Jutiapa from Chiquimula, Guatemala (Fig 9). Infected specimens from all sites were clustered around the origin. Permutation tests determined three statistically significant clusters: (1) non-infected specimens from Jutiapa, Guatemala (p = 0.031), (2) non-infected specimens from Chiquimula (p = 0.028), and (3) infected-specimens from multiple locations (p = 0.043) (Fig 9).

### Blood meal detection

**Fig 10:**
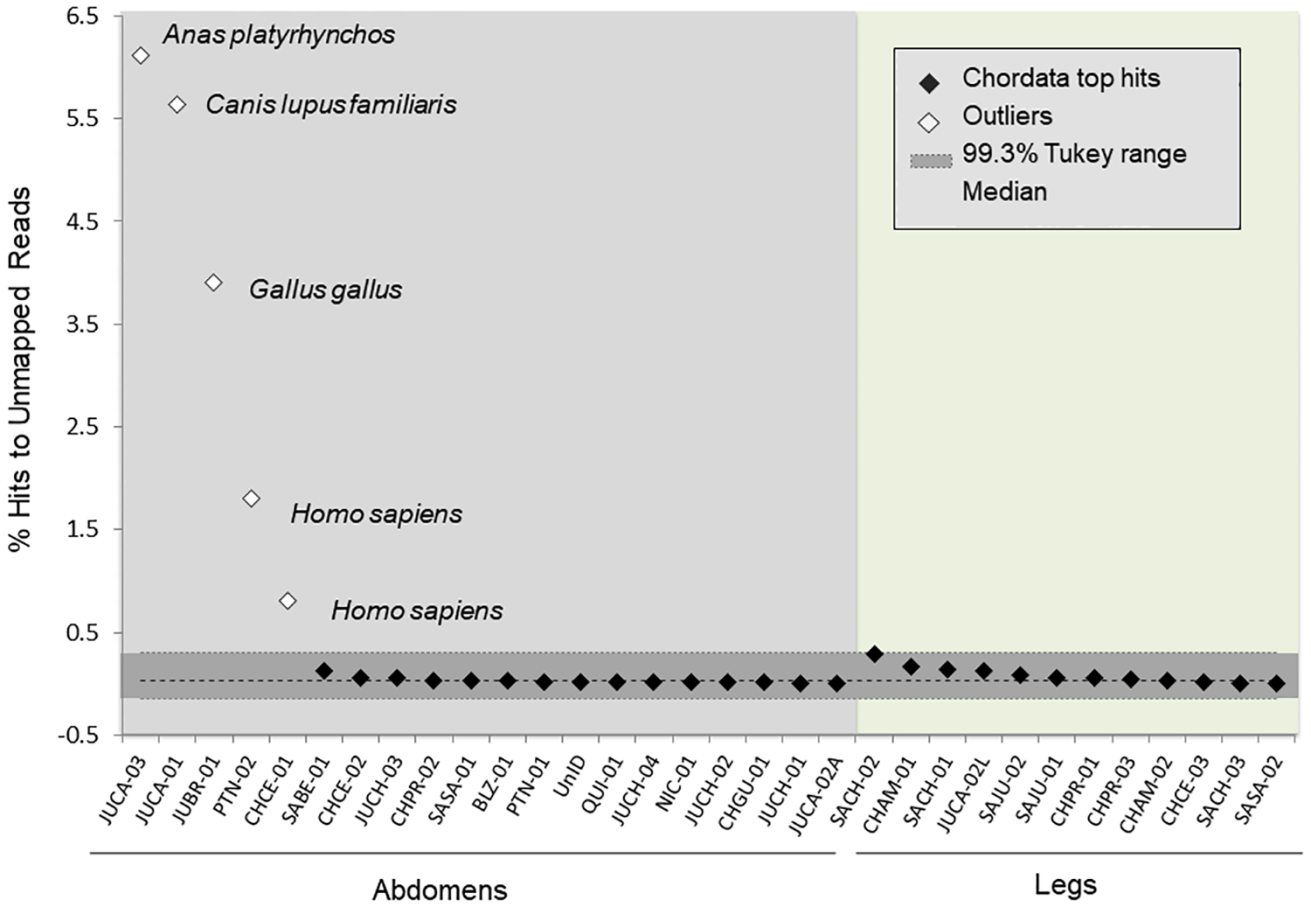
Outlier test of top chordate hits. Specimens are sorted by top-hit percentage; legs were included in the analysis as baseline controls. Species identity of the top hit is indicated for the five abdomens above the upper Tukey range (+1.5*IQR).

Five abdomens returned chordate reads for a single top-hit species at an order of magnitude higher than the background threshold calculated from the leg controls. Top hits for these specimens included chicken, dog, duck and human (Fig 10). Reads that matched chordates were present in all 32 specimens, including both abdomens and legs. The top hits had an exceedingly low representation in most specimens (median = 0.035% of reads; Fig 10); these included human (n = 23), domestic birds (chickens and ducks) (n = 5), dog (n = 1), fish (n = 1), ruminant (n = 1) and frog (n = 1) (S5 Table).

## Discussion

Our results suggest that RADseq can be used to simultaneously investigate *T*. *cruzi* infection and phylogenetic reconstruction of DTUs, population genetic structure of *T*. *dimidiata*, parasite-microbial interactions in the gut of the vector, and predominant blood meal source. For vector-borne diseases that involve multiple interacting species, methods that can produce data on an entire community can be used to leverage a single genetic study to address multiple biological questions across a range of taxa. Although the approach has some limitations, there was sufficient information to identify biologically meaningful patterns of genetic and community structure at a range of spatial scales, from individual villages to across Central America. Furthermore, the modest minimal requirements of 2–3 million reads to recover sufficient data on all taxa also makes RADseq a relatively economical method, with expected sequencing costs in 2016 of ~$30/specimen using current sequencing technologies (e.g., HiSeq 2000). Notably, the method can be successful even for specimens preserved for considerable periods prior to sequencing, although careful assessment of DNA quantity and quality is critical for recovering sufficient high-quality read information from target taxa.

RADseq successfully identified *T*. *cruzi* infection across multiple DTUs (Fig 5), with higher sensitivity than microscopy. The sensitivity of the method is important for surveys of parasite prevalence in natural populations, as *T*. *cruzi* infection intensity within vectors can range from high to exceedingly low representation of the parasite in the hindgut, and can vary across populations, species, physiological condition of the vector, anti-microbial activity in the gut and haemolymph, and co-occurrence of other pathogens and symbionts [67, 68, 69]. In general, molecular methods such as PCR-based detection have proven more sensitive compared to microscopy, but replicability of PCR methods is dependent on the volume of parasitic DNA extracted from the hindgut, the extraction protocol, and the DNA region that the probes amplify [70, 71]. Given the low representation of the parasite across all specimens (1% of all trimmed reads), *T*. *cruzi* is likely to be more readily detected in RADseq libraries prepared with longer restriction enzymes that cut in fewer recognition sites, allowing higher depth of coverage across the parasite genome (6–8 bases, e.g. *SbfI* or *PstI*). Careful dissection to maximize the representation of parasite-rich tissues such as the lower abdomen and anus may also assist in *T*. *cruzi* recovery by preventing overrepresentation of the vector during sequencing.

When *T*. *cruzi* is found, the genome-wide sampling provided by RADseq, in combination with the availability of reference genomes, also provides an effective tool for *T*. *cruzi* DTU identification and phylogenetic reconstruction. The two DTUs identified among the nine infected specimens clustered into two clear clades, with strong bootstrap support and branch lengths between clades ~10-fold longer than that within each DTU (Fig 6). The more common of these closely matched TcI, the DTU expected to be the most common in circulation in Central America [7, 11, 13]. The identity of the second DTU is unclear, as it did not cluster with any of the DTUs for which sequenced reference genomes are available. The two DTUs most commonly found in Central America are TcI and, less frequently TcIV, for which a reference genome was not available (previously TcIIa) [72–78]. As additional references become available, the power of the RADseq mapping approach to positively assess DTU identities throughout the Americas should progressively increase.

Despite the absence of a sequenced reference genome for *T*. *dimidiata*, this study effectively identified SNP markers useful for understanding vector population structure. Even with relatively strict filtering criteria, using a small set of vector-only reference specimens to create a species-specific catalog yielded tens of thousands of SNP markers (Figs 2 and 4a), and the low BLAST mapping to other insects (0.06% of all trimmed reads) suggests that the method captured a substantial proportion of the true *T*. *dimidiata* tags in the mixed-DNA specimens. The SNP dataset was sufficiently large to enable population-genetic analysis across spatial scales with a single methodology, with thousands of variable loci present within individual villages that increased with each successive increase in spatial scale included (Table 3). Such flexibility is a considerable advantage over traditional markers, such as microsatellites or multi-locus gene sequencing, which are each most appropriate for questions at a particular temporal or spatial scale but uninformative for others. Even with the limited sampling included here, patterns of allelic variation successfully resolved biogeographic structure at multiple geographic scales (Fig 7), yielding four distinct genetic clusters corresponding to departmental and regional geographic divisions. As in previous studies, our results suggest moderate levels of differentiation within *T*. *dimidiata* across this region of Central America [24, 26, 27], although clearly more comprehensive sampling focused on thorough biogeographic coverage will be needed to evaluate these patterns further.

Although informative SNP markers were identified across all villages and departments in the present study, genetic variability was not consistent across space, with a range of 9–30% of loci showing polymorphisms at the village scale for samples that in all cases but one were collected in the same year for each village and with similar sample sizes (Table 3). This likely represents underlying variation in genetic diversity across the range of *T*. *dimidiata*; it is important to note that the current study focused on a portion of the species’ range, and thus it is not clear whether the variation and genetic structuring suggested here will extend to other regions or vector species. Even when variability was relatively low, however, the scale of genomic coverage afforded by techniques such as RADseq yielded a large absolute number of SNPs from the perspective of population-genetic analysis, and thus should facilitate effective SNP discovery for all but the most genetically uniform populations and species.

RADseq can also reveal biologically interesting comparative patterns of microbiome variation that can subsequently be explored with more in-depth metagenomic approaches. From this study, two main drivers of gut bacterial community structure are evident. First, bacterial communities were strongly locally structured, with distinct species assemblages even between Jutiapa and Santa Ana, whose vector populations are not differentiated (Figs 7 and 9). Whether this is true spatial patterning, or reflects temporal, seasonal or other environmental variation among sites at the point of sampling or during processing cannot be determined from these data; however, this is an interesting avenue for future research. Second, *T*. *cruzi* parasitic infection significantly increases the diversity of bacteria (p <0.01), introducing a common additional set of infection-associated microbiota across the entire region (Fig 9). These patterns are consistent with recent literature demonstrating shifts in bacterial diversity across vector genera, by geographic location, and parasitic infection status [35, 79]. How *T*. *cruzi* interacts with gut microbes is a promising area of future research in this system, as infection prevalence is highly variable across Central America and may be affected by the ability of native microbial communities to resist colonization [40, 79]. Further studies of infection-associated bacterial taxa may also reveal important aspects of the transmission cycle. Infection may facilitate bacterial colonization due to modification of the immune response of the vector or changes in the gut lining [33, 38]; alternatively, successful infection may be the end result of bacterial compositional changes associated with insect condition, health or other factors that make the gut environment more favorable for *T*. *cruzi* attachment [32, 34, 35, 36, 37, 41, 79].

Although RADseq can identify community patterns, it is likely to be poor for species-level identification of individual taxa such as bacterial symbionts that are not anticipated *a priori*. True species identity often could not be ascertained with confidence due to database limitations and lack of sequence specificity; a significant drawback of RADseq is the short read length, which can make it difficult to assign taxonomic identity with precision. Of the set of reads that did not map to either the parasite or vector, significant BLAST hits were returned for 20.1% of the queried reads (Fig 3a). Even in the subset of reads with a significant hit, the likelihood that the taxon returned was the true DNA source depended on its representation in the nt database as well as the degree of evolutionary conservation of the genomic region. This was most evident in reads assigned to chordates, which occasionally returned species that clearly were not locally available, including model organisms (e.g., zebrafish) and Old-World relatives of putative blood meals (e.g., gorilla). These were rare (~1%), and appear to represent highly conserved loci with close matches to a diverse set of taxa; because species calls were made without regard to how much better the top hit matched the query than the subsequent taxa; loci with equally-close matches to multiple taxa returned results that were consistent across runs but essentially arbitrary with respect to the species listed first. It is more difficult to assess the degree to which misassignment occurred in other taxonomic groups.

With an undirected sequencing approach like RADseq, sequencing reads from the gut microbiome are an automatic consequence of targeting tissues harboring *T*. *cruzi*. Whether RADseq is sufficient for answering microbial community questions, however, is likely dependent on the type of information required. If the goal is to identify species that interact with *T*. *cruzi* or influence its transmission (e.g., *Serratia marescens* [38]) or produce novel or functionally important chemical compounds, alternative next-generation sequencing methods such as shotgun metagenomic, transcriptomic and/or meta-barcoding methods could provide higher specificity and quantitative precision. This is less of a critical issue for community composition analysis, however, because the Unifrac procedure incorporates phylogenetic relationships into the distance measure, linking specimens even when minor sequence differences lead to different species calls.

Given that (1) triatomines can live for several months in starvation, (2) the vast majority of insects sampled here were adults, which ingest proportionally smaller blood meals than nymphs, and (3) many field studies have found that specimens are often starved at the moment of collection, it was not surprising that we were able to confirm putative sources of blood meal from just 25% of the abdomens analyzed [80–83]. Nevertheless, the fact that contamination from human handling was uniformly present across samples, the RADseq approach was arguably least effective at resolving vector-feeding patterns, and is likely to be useful only for very recent or large blood meals. Minimizing handling, along with surface-sterilizing and extracting DNA under sterile conditions are advisable for minimizing such sources of ambiguity.

In addition to background contamination, the strict DNA quality requirements for next-generation sequencing technologies likely introduce biases against detecting blood meals. Although using abdomen DNA has the tremendous advantage of investigating mixed taxa, the use of abdomens presents the challenge of obtaining high-quality DNA that has not been degraded by digestion. Previous studies targeting blood meals using species-specific primers recommended the use of PCR-based assays targeting small size amplicons of nuclear DNA to detect unique blood meals instead of a catchall method [82–84]. In our experience, obtaining high-quality DNA from the hindgut of adult *T*. *dimidiata* was challenging, with a total of 61 insects required to obtain the final 32 specimens. Even among these specimens, sequencing yield ranged from 489,656 to 18,878,597 reads, a 38-fold range. Many DNA specimens excluded from sequencing were characterized by a strong second band of degraded DNA at 100-200bp, possibly a degraded blood meal, in addition to the expected high-molecular weight band (S1 Fig). The degradation from blood meal digestion is compounded by the challenge of field preservation, storage, and transport of specimens from remote areas with limited infrastructure. Although not enough specimens were tested to allow statistical comparisons, higher extraction success tended to be achieved when specimens were collected closer to the extraction date than those collected 3+ years earlier. Additionally, the time delay between DNA extraction and sequencing was kept to a maximum of one month to maintain the quality of the specimens and avoid DNA degradation during storage.

A benefit of using taxonomically, non-specific sequencing approaches like RADseq is the potential for discovery of unexpected taxa that may be of ecological or epidemiological importance. One such finding was the common presence of entomopathogenic fungi (22% of fungi hits). Although none of the specimens showed visual evidence of cuticular fungal germination, the presence of *Beauveria*, *Metarhizium*, *Zoophthora*, and *Entomophaga*, both in the abdomens and legs, suggest possible latent infection of the vectors by spores waiting for environmental cues that can trigger germination [85]. Although the fungal inoculation sources are unknown, the presence of the entomopathogenic genera across tissues and specimens suggests a wide distribution of spores regardless of the local environment in which the triatomine was collected [86].

We also found a low signal of entomopathogenic nematode species from the family *Steinernematidae*. Additionally, the BLAST search revealed a wide range of common mammalian parasitic nematodes from the genera *Angiostrongylus*, *Heligmosomoides*, *Haemonchus*, *Parastrongyloides* and *Strongyloides* (Fig 2). Although to some extent this may be a result of transfer from humans to the bug during handling, this result raises the possibility that *T*. *dimidiata* may harbor and/or transmit such parasites as a passive carrier of infective free-living larvae or eggs [87]. This is a meaningful finding because of the potential of co-transmission of additional human pathogens, which has been previously documented in other vectors such as *Aedes aegypti* and *A*. *albopictus* [88]. The role of a triatomine vector could either involve the cutaneous transportation of the nematode as it moves from dirt crevices to the skin of mammalian host or by gut transportation; eventually defecating eggs near open wounds, eyes, or areas prone to oral contamination [89, 90]. It is unlikely that the vector can acquire the nematodes from a blood meal source given that only the genus *Strongyloides* is known to have a non-reproductive larval stage in the human bloodstream, and even in this case, it is cutaneously transmitted, remaining in the bloodstream only in transition to the small intestine [91]. The detection of other human pathogenic nematodes opens new avenues of research to study the role of triatomines in the context of vector-aided transmission. Although the aim of this study was not to reveal community patterns beyond the parasite, vector and microbiota, our findings can potentially lead to community-based studies of entomopathogenic fungi and nematodes, human parasitic nematodes and other taxa with relevant association to disease transmission complexes.

## Conclusions

Overall, our results show that a mixed-DNA approach can provide simultaneous information on the community of biotic factors involved in *T*. *cruzi* transmission. RADseq can provide informative SNP marker sets for taxonomic and biogeographic analysis for both vector population genetic structure and parasite evolutionary history. It also has a strong potential to retrieve information about the community ecology and diversity of microbiota; and although it is limited at revealing quantitative details of vector feeding history, this method may be useful for identifying recent vertebrate hosts. For all of these areas of inquiry, a broad-based sequencing approach can reveal novel patterns that can be followed up with complementary approaches (e.g., proteomics, metagenomics). Testing this mixed-DNA sequencing method with different vectors and disease models will help to determine its reproducibility in other systems where multiple organisms interact in tightly-integrated and complex ways.

## Supporting information

S1 Fig Representative agarose gel separation of extractions from abdomen of *T*. *dimidiata*. The upper band, indicated by the black arrow, is intact genomic DNA. In lanes 6–9, 12, and 13–14, a second band of degraded DNA (1-200bp), indicated by the red arrow, is likely partially-digested blood meal. 1kb ladder is loaded in the far-right lane for comparison.
(DOCX)

S1 Table BLAST e-values by taxonomic group. (XLSX)

S2 Table *T*. *cruzi* DTU descriptive statistics. (XLSX)

S3 Table Bacterial counts per sample. (XLSX)

S4 Table Blood meal BLAST descriptive statistics. (XLSX)

S5 Table Individual F-statistics. (XLSX)
