# Supplementary material for: Uncovering vector, parasite, blood meal and microbiome patterns from mixed-DNA specimens of the Chagas disease vector Triatoma dimidiata
Source: PLoS Negl Trop Dis. 2018 Oct 18;12(10):e0006730. doi: 10.1371/journal.pntd.0006730 (PMC6193617; doi:10.1371/journal.pntd.0006730)
Supplement: S1 Fig — The upper band, indicated by the black arrow, is intact genomic DNA. In lanes 6–9, 12, and 13–14, a second band of degraded DNA (1-200bp), indicated by the red arrow, is likely partially-digested blood meal. 1kb ladder is loaded in the far-right lane for comparison. (DOCX) [file pntd.0006730.s001.docx]

Gel concentration: 1.5%

Voltage: 120 V

Time: 50 min

DNA V loaded per sample: 4 uL

Dye V loaded per sample: 2 uL


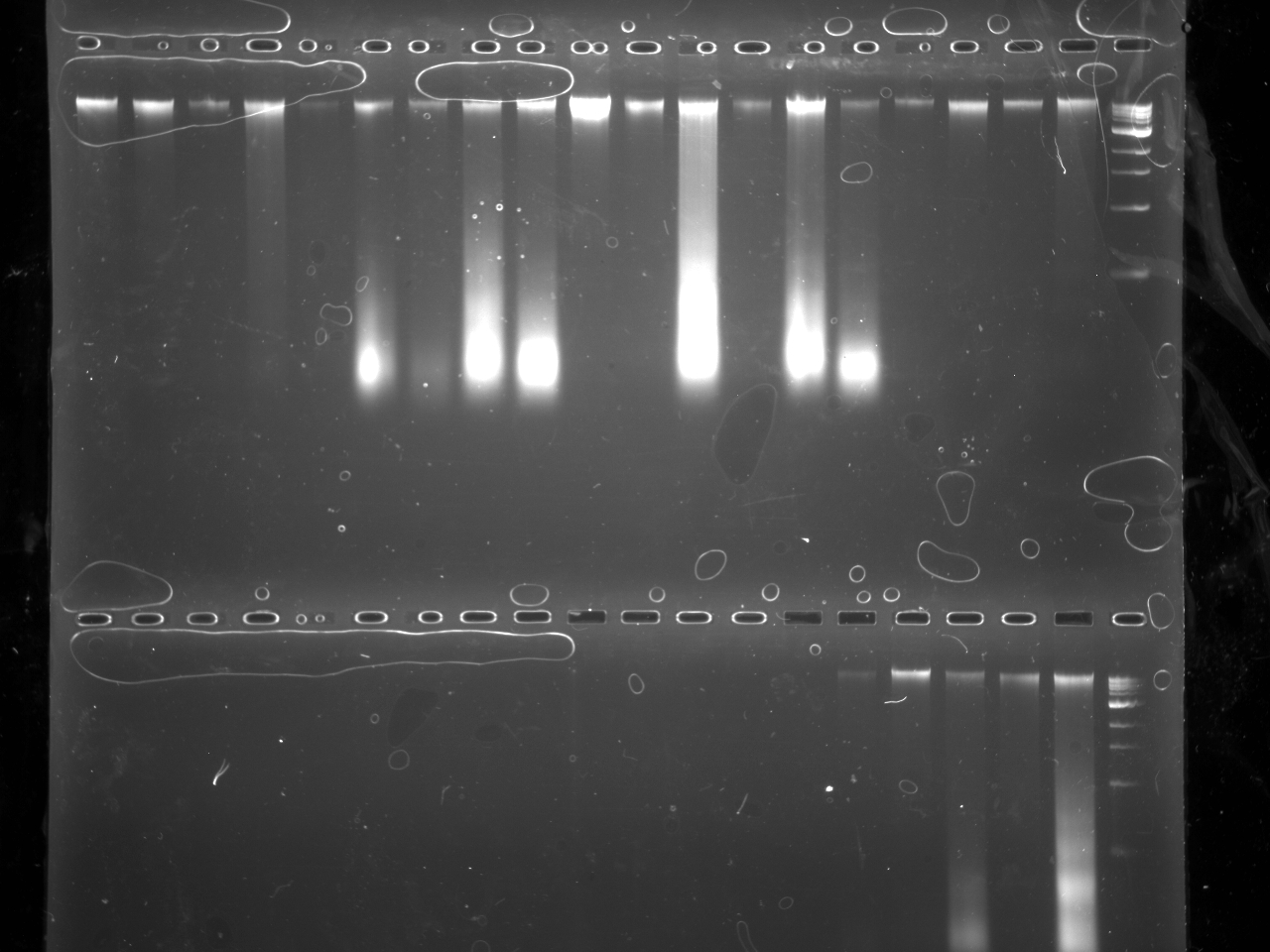


**Fig. S1** representative agarose gel separation of extractions from abdomen of T. dimidiata. The upper band, indicated by the black arrow, is intact genomic DNA. In lanes 6-9, 12, and 13-14, a second band of degraded DNA (1-200bp), indicated by the red arrow, is likely partially-digested blood meal. 1kb ladder is loaded in the far-right lane for comparison.
